# The Bacterial Composition within the *Sarracenia purpurea* Model System: Local Scale Differences and the Relationship with the Other Members of the Food Web

**DOI:** 10.1371/journal.pone.0050969

**Published:** 2012-12-05

**Authors:** Sarah M. Gray, Denise M. Akob, Stefan J. Green, Joel E. Kostka

**Affiliations:** 1 Department of Ecology and Evolution, Stony Brook University, Stony Brook, New York, United States of America; 2 Unit of Ecology and Evolution, Department of Biology, University of Fribourg, Fribourg, Switzerland; 3 Institute of Ecology, Friedrich Schiller University, Jena, Germany; 4 DNA Services Facility, Research Resources Center, University of Illinois at Chicago, Chicago, Illinois, United States of America; 5 Department of Biological Sciences, University of Illinois at Chicago, Chicago, Illinois, United States of America; 6 Georgia Institute of Technology, Schools of Biology and Earth & Atmospheric Sciences, Atlanta, Georgia, United States of America; Argonne National Laboratory, United States of America

## Abstract

The leaves of the carnivorous pitcher plant, *Sarracenia purpurea*, contain a microscopic aquatic food web that is considered a model system in ecological research. The species identity of the intermediate and top trophic level of this food web, as well the detritivore midge, are highly similar across the native geographic range of *S. purpurea* and, in some cases, appear to have co-evolved with the plant. However, until recently, the identity, geographic variation, and diversity of the bacteria in the bottom trophic level of this food web have remained largely unknown. This study investigated bacterial community composition inside the leaves of *S. purpurea* to address: 1) variation in bacterial communities at the beginning of succession at the local scale in different areas of the plant’s native geographic range (southern and mid-regional sites) and 2) the impacts of bacterial consumers and other members of the aquatic food web (i.e., insects) on bacterial community structure. Communities from six leaves (one leaf per plant) from New York and Florida study sites were analyzed using 16S ribosomal RNA gene cloning. Each pitcher within each site had a distinct community; however, there was more overlap in bacterial composition within each site than when communities were compared across sites. In contrast, the identity of protozoans and metazoans in this community were similar in species identity both within a site and between the two sites, but abundances differed. Our results indicate that, at least during the beginning of succession, there is no strong selection for bacterial taxa and that there is no core group of bacteria required by the plant to start the decomposition of trapped insects. Co-evolution between the plant and bacteria appears to not have occurred as it has for other members of this community.

## Introduction

The carnivorous perennial pitcher plant, *Sarracenia purpurea*, native to wetlands and bogs throughout the eastern United States and most of Canada, provides an excellent natural laboratory in which to address the role that bacterial communities play in aquatic food web dynamics. The leaves of *S. purpurea* collect rainwater after opening, providing a micro-environment for an aquatic food web to develop [1], [2]. These spaces are referred to as phytotelmata, or plant-held bodies of water, and are common and widely studied naturally occurring micro-habitats. The community dynamics within these leaves are similar to those of larger aquatic food webs, but on small spatial and short time scales (e.g.) [2], [3]. The pitcher plant has been developed as a model system to test fundamental questions in community ecology including the importance of top-down and bottom-up forces in structuring communities, and the potential for trophic cascades [3], community consequences of invasion [4], nutrient limitation [5] and commensalisms [6].

The development of the aquatic food web held by the leaves of *S. purpurea* begins when newly opened leaves collect rainwater and act as a pitfall trap for insects. Captured insects provide nutrients for the plant and become the basal resource for the food web. Once insects have drowned in the water, aquatic invertebrates that live within the pitcher break down the dead insects into smaller fragments and bacteria decompose the insects, releasing nutrients that can then readily be taken up by the plant. Unlike other species of pitcher plants, *S. purpurea* does not produce digestive enzymes, except possibly in newly opened leaves [7], and is therefore largely reliant on bacteria to decompose the captured insects. The degree to which bacteria can decompose insects is dependent on the abundance of protozoans and rotifers in the intermediate trophic level, which feed on the bacteria, and on the presence of the top predator (larvae of the endemic mosquito *Wyeomyia smithii* (Culicidae), which feeds primarily on the protozoans and rotifers, but also occasionally on bacteria [3], [8]. The midge *Metrocnemus knabi* (Chironomidae), found along the bottom of a pitcher, facilitates the release of nutrients into the food web by breaking the dead insects into smaller pieces [6].

Because of the close association between bacteria and the plant, it can be hypothesized that specific bacterial species might be particularly important (e.g., those involved in chitin degradation, protease and other extracellular enzyme production), and this subset of bacteria should be expected in high frequencies in leaves throughout the geographic range of the plant. This pattern is already found with the rotifer, common protozoans, and dipteran species present in this community, which are believed to largely be the same set of species throughout the plant’s broad native geographic range [2], [9], [10]. Few studies have investigated bacterial communities in pitcher plants and fewer still were conducted using cultivation-independent molecular approaches. For decades, culture studies have shown that free-living bacteria are key members of the aquatic community in the pitchers of *Sarracenia* plants (e.g.) [3], [5], [8], but specific microbial groups were rarely identified. Recently, using a DNA fingerprinting approach, Peterson et al. [11] took the first step towards characterizing the variability in bacterial community composition in microbial biofilms from *S. purpurea* leaves in central Massachusetts bogs. Krieger and Kourtev [12] followed by identifying the bacteria of the three sub-habitats (bottom sediment, liquid, and leaf wall) within *S. purpurea* pitchers from Michigan bogs. In addition, Koopman and colleagues used high throughput genetic techniques to provide a detailed characterization of bacterial community composition in leaves of the cogener, *S. alata* [13], [14].

The study presented here used 16S ribosomal RNA (rRNA) gene sequencing to identify the bacterial composition within *S. purpurea* leaves at the beginning of succession at two different sites in the geographic range of this plant species, Florida and New York. This information, in addition to that from other published work (e.g.) [11], [12], help to shed light on if, like the other members of the *S. purpurea* food web, specific bacteria are found in this system and if so, what type of bacteria they are. In addition, this study places the bacterial community composition into context with all the remaining members of the aquatic food web that inhabit the leaves of *S. purpurea.* This is a novelty not yet addressed with this system and will help in understanding the structure-function relationships of bacterial communities in pitchers. Relating the identification of specific bacterial groups in the pitchers of *S*. *purpurea* with the diversity and relative abundance of insect larvae, rotifers, and protozoans that make up the remainder of the food web is essential for contributing to the understanding of the interplay between food web complexity and structure with food web dynamics.

## Methods

### Ethics Statement

All necessary permits were obtained for the field sites used in this study. For the Long Island site, the permit was issued by the Suffolk County Department of Parks. For the Florida site, the permit was issued by the Apalachicola Ranger District in the United States Department of Agriculture.

### Study System, Field Site and Sampling

*S. purpurea* is native to nutrient-limited wetlands and bogs throughout the eastern United States and most of Canada, with the southernmost population located in the panhandle of northern Florida. *S. purpurea* is composed of two subspecies: subspecies *purpurea* (north of New Jersey, including New York) and subspecies *venosa* (south of New Jersey) [15]. The population located in north Florida is thought to be a separate variety, var. *burkii* [16], but contain the same members of the aquatic community as are seen throughout North America [10], [17]. The leaves form in a rosette pattern, with the main morphological difference between the subspecies being the shape of the leaf (diameter of the opening of the pitcher, wing size, and size and frill of the hood vary in size according to subspecies) [15]. The phytotelmata of all subspecies also harbor the same insect larvae, rotifer, and common protozoan species throughout the plant’s geographic range [10], [17].

Pitcher plant populations in a bog in Sumatra, Florida (Apalachicola National Forest) and a bog near Riverhead, New York (Cranberry Bog Preserve) were chosen for this study. These sites represent the mid-range (New York) and southern range (Florida) of *S. purpurea*. Although *S. purpurea* is found in nitrogen poor soils, characteristics of the local habitat can vary greatly. The New York bog site used in this study is composed of sphagnum moss and contains only one other carnivorous plant species, the roundleaf sundew *Drosera rotundifolia,* which grows on the sphagnum moss alongside cranberry (*Vaccinium macrocarpona)* shrubs and the reed *Phragmites australis*. The most common ant species (basal resource) found within the leaves of *S. purpurea* plants at this field site is *Tapinoma sessile* [18].

The Florida bog site is located in a sandy, open savannah within a long leaf pine (*Pinus palustris*) forest. The habitat is mainly composed of the grass *Aristida stricta* and a large diversity of other carnivorous plant species (*S. flava, S. psitticina, Drosera capillaris* and *Pinguicula* species). The most common ant species (basal resource) inside the pitcher plant leaves at this field site is the invasive fire ant *Solenopsis invicta* [3].

At the beginning of the growing season for each location (May 2008 for Florida and June 2008 for New York), the first fully developed yet unopened leaves of that season were marked. These plants were randomly selected based on walking through the bog and marking unopened leaves. Within two weeks of being marked, each leaf opened into its characteristic pitcher shape, filled with rainwater, collected insects into its pitfall trap, and the aquatic community of protozoans, rotifers, bacteria, and larvae assembled in the pitcher. After 14 days, 15 leaves in Florida and 15 leaves in New York (one leaf per plant) were selected for further analysis. The members of the entire aquatic community (detritus, culturable bacteria, protozoan and rotifer species and insect larvae) as well as the water volume and clarity were characterized for all 15 leaves at the two sites. Six of these samples at each site (6 in NY and 6 in FL) were used to develop 16S rRNA gene clone libraries to assess bacterial community composition. The water in each selected leaf was gently mixed with a sterile pipette and placed into a sterile 50 ml centrifuge tube, which was transported on ice back to the laboratory for further processing.

### Processing of Pitcher Plant Aquatic Community

We assessed the richness and abundance of the common members of the pitcher plant aquatic community according to standard methods (e.g.) [3], [5]. The volume and clarity of the water was recorded for each sample and the number of dead ants and other invertebrates present in the water were counted. We used a compound microscope to determine the richness and densities of protozoan species within a 0.1 ml aliquot of each pitcher plant aquatic community sample. To determine the relative abundance and richness of the culturable bacterial morphotypes, a 10^−4^ dilution of the pitcher plant water was plated onto a half-strength Luria broth plate [5], [8]. Plates were incubated at 26°C for 72 hours after which colony forming units (CFUs) were counted [3], [5].

### Environmental Clone Library Construction and Phylogenetic Analyses

A 1 ml aliquot of the water sample from each pitcher was filtered onto a 0.22 µM Isopore membrane filter (Millipore, Billerica, Massachusetts) to collect bacterial cells. Microbial community DNA was extracted from the filters using the Ultra Clean Soil DNA kit according to manufacturer’s instructions (Mo Bio Laboratories, Solana Beach, California), with the exception of Step 1 of the protocol. Instead of using a soil sample, the filter was placed in the bead solution tubes with 200 µl of sterile water.

Aliquots of purified DNA were PCR amplified using the *Bacteria* domain-specific 16S rRNA gene primers 27F (5′-AGA GTT TGA TCM TGG CTC AG -3′) [19] and 1392R (5′-ACG GGC GGT GTG TAC-3′) [20] as previously described [21]. PCR products were purified using the Qiagen Gel Extraction Kit (QIAGEN, Valencia, CA), then ligated into the TOPO TA cloning vector pCR 2.1 according to manufacturer’s instructions (Invitrogen, Carlsbad, CA). Ligation reactions were transformed at The Genome Center at Washington University (St. Louis, Missouri). Clones from New York libraries were sequenced using the primers 27F, 907R and 1392R and the Florida clones were sequenced in a single direction with primer 907R.

Sequences were assembled and vector sequences flanking the 16S rRNA gene inserts were removed using Sequencher v4.8 (Gene Codes Corp., Ann Arbor, MI). Sequences were aligned using the Greengenes alignment tool [22] and were then imported into the Greengenes sequence database [23] within the phylogenetic software package ARB (http://www.arb-home.de; [24]). Partial sequences recovered in this study were inserted into the Greengenes 16S rRNA gene tree using the ARB parsimony option, employing a Bacterial 50% conservation filter [25], while the overall topology of the tree was maintained. The phylogenetic tree was exported from ARB and analyzed using the online software package Unifrac [26]. Sequences were also submitted to the Ribosomal Database Project (RDP) Classifier [27] to generate taxonomic classification to the genus level. Diversity and community composition analyses were performed on sequence data classified to the genus level. Sequence data were deposited in the EMBL database under accession numbers HE820984 − HE821226 and HF544513 − HF544978.

### Variation in Community Composition and Diversity Indices

To accurately assess diversity patterns between leaves, the number of clones was equalized for each clone library by randomly selecting 49 clones from each sample. We then calculated diversity indices (Shannon Index and richness) as well as Pielou’s evenness with the software package Primer 6 (Version 6.1.6, Primer E-Ltd. 2006). Subsequently, nonparametric multivariate statistics, implemented within the software package Primer 6 and based on genus-level identification of sequences performed by the RDP classifier, were used to determine the similarity in bacterial community structure at the local scale within and between the two sites. For similarity in abundances, data were first normalized using a square root transformation. Bray-Curtis distances were then calculated, which uses values from 0 (most similar) to 1 (least similar) to determine similarity between samples [28]. To graphically visualize the differences between bacterial communities, a non-metric Multi-Dimensional Scaling (MDS) plot was used. Communities that are more similar are spatially close to each other on a MDS plot and those that are less similar are spatially separated. An Analysis of Similarity (ANOSIM) was performed to calculate a Global R, which determined the overall similarity between communities, with a value of 1 representing extreme dissimilarity and a value of 0 representing complete overlap in community composition. To determine if results based on the discrete data generated by RDP genus-level identification were robust, ANOSIM results and MDS plots were also compared to those generated from data obtained from the Unifrac measure based on continuous phylogenetic data generated from ARB.

## Results

### Variation in Bacterial Communities within and between Bogs

**Figure 1:**
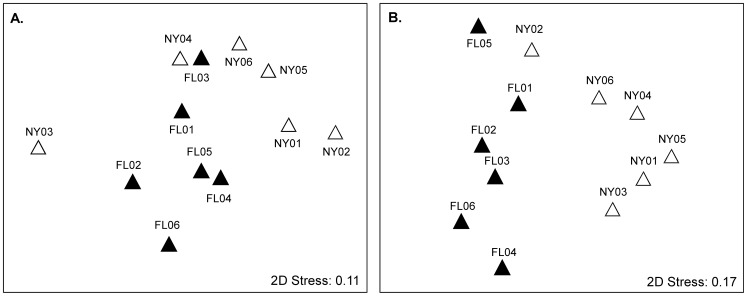
Beta-diversity of bacterial communities present in leaves of *S. purpurea* from NY and FL sampling locations using Bray-Curtis dissimilarity. MDS plots comparing bacterial community structure within sampling sites and among leaves were generated based on organismal classification based on colony morphology of culturable bacteria (A) and 16S rRNA gene sequences, identified at the genus-level using the RDP classifier (B). Data were based on square-root transformed Bray-Curtis similarity. Each symbol represents the bacterial community in one pitcher plant leaf (FL = Florida; NY = New York).

The similarity in bacterial community composition between pitcher plant leaves within and between sites when only culturable bacteria are considered (agar plate counts) and when bacterial genus-level identification (based on 16S rRNA gene sequences) is considered are shown in multidimensional scaling plots (Figure 1a, b). This graphical representation of community similarity was performed for the abundance of individual morphotypes (Figure 1a) and the abundances of individual genus-level identification (Figure 1b). The resulting MDS plots illustrate that for both culturable bacteria and genus-level identification, bacterial communities both within a site and between sites are significantly different from one another, and bacterial communities within a site are more similar to each other than when compared across sites (Figure 1a, b). This result was especially the case for the abundances of genera obtained from sequence data, in which composition within a site was more similar than composition between sites (Figure 1b; ANOSIM Global R = 0.596, p-value = 0.002. There was also a similar, but weaker, pattern of overlap for culturable bacteria composition (Figure 1a, ANOSIM Global R = 0.306, p-value = 0.02).

**Figure 2:**
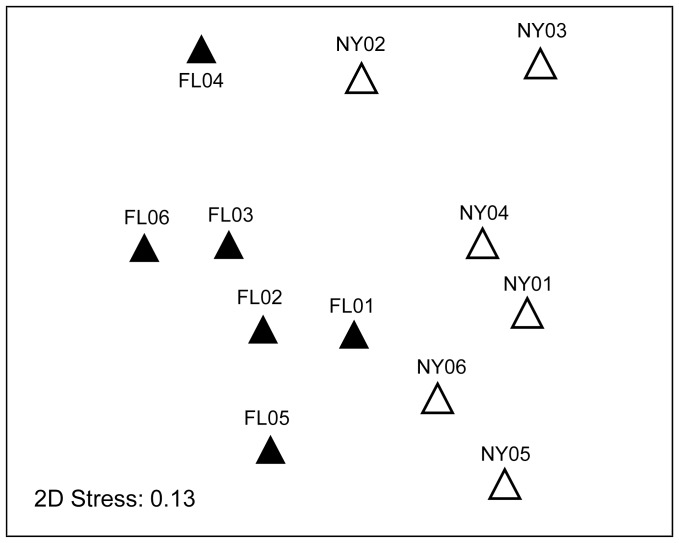
Beta-diversity of bacterial communities present in leaves of *S. purpurea* from NY and FL sampling locations using the Unifrac metric. Bacterial community structure was assessed by sequencing 16S rRNA genes as described in the text. A phylogenetic tree was generated by inserting partial gene sequences recovered in this study into a tree based on near-full length sequences, implemented within the software package ARB. The phylogenetic tree was analyzed using the software package Unifrac, and a pair-wise distance matrix was generated for comparison of the bacterial community in each leaf. This matrix was used to generate a MDS plot, demonstrating distinct bacterial communities in FL and NY leaves.

These findings were corroborated using the clustering-independent Unifrac analysis. The resulting ANOSIM from the Unifrac analysis showed that samples from FL were more similar to each other than to samples in NY (Figure 2). We observed that the ANOSIM Global R value was smaller (R = 0.474, p-value = 0.004) between New York and Florida samples using Unifrac than for the RDP-based methods, which is consistent with the reduced phylogenetic information present in genus-level identification data.

**Table 1:** Statistical analyses of 16S rRNA gene sequence libraries using ecological estimates of sequence diversity.

Location	Leaf	Number of clones	Number of genera	Shannon-Wiener (H′)	Pielou’s evenness
New York	1	49	14	2.45	0.93
	2	49	12	2.36	0.951
	3	49	16	2.66	0.96
	4	49	19	2.83	0.962
	5	49	14	2.5	0.946
	6	49	12	2.33	0.939
Florida	1	49	11	2.21	0.921
	2	49	18	2.77	0.96
	3	49	16	2.7	0.973
	4	49	9	1.99	0.91
	5	49	12	2.33	0.94
	6	49	11	2.29	0.954

There was no difference in diversity and evenness between the FL and NY sites.

Culturable bacterial diversity and evenness was not significantly different when pitchers sampled in New York were compared to pitchers sampled in Florida (Mann Whitney U Test, Diversity: p = 0.309, Evenness: p = 0.443) or when sequence data classified into genera were analyzed (One-way ANOVA, Diversity: p = 0.354, Evenness: p = 0.623; Table 1).

**Figure 3:**
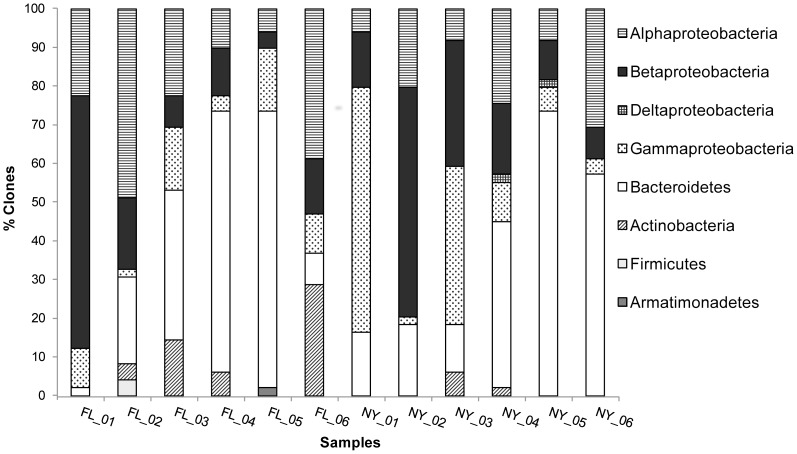
Frequencies of bacterial phylogenetic lineages in *Sarracenia purpurea* water from Florida and New York. Calculations were made based on the total number of sequences in each 16S rRNA gene library associated with a single taxon.

Grouping based on RDP classification detected a total of 93 genera in the entire data set, with 52 genera detected in the New York leaves and 47 genera detected in leaves from Florida. Eighteen genera were detected in leaves from both sites, though the abundance differed substantially between the two sites (Figure 1). Although additional sampling of clones would be necessary to describe the overall diversity fully, sequences from dominant bacterial genera were obtained. Results from the RDP database showed that most sequences were predominately closely related to uncultivated environmental bacteria and belonged to the phyla Proteobacteria and Bacteroidetes (Figure 3). In addition, 5 genera belonged to the phyla Actinobacteria, 2 belonged to Firmicutes, and 1 belonged to Armatimonadetes (Figure 3).

Members of the phylum Proteobacteria were detected in aquatic communities from both Florida and New York leaves; however, not all subclasses were detected at both sites. Members of the Alpha-, Beta-, and Gamma-subclasses of the Proteobacteria were detected at both sites and in all leaves, whereas, the Deltaproteobacteria subclass was only found in New York leaves 4 and 5. Furthermore, according to the RDP results, the most dominant genera in one site were not the most dominant genera present in the other site. For Alphaproteobacteria, the most dominant genus detected in Florida leaves was *Sphingomonas* represented 17% of all FL sequences), but in New York the most common genera were *Raoultella* (10% of all NY sequence), *Rhizobium* (8% of all NY sequences), and *Pseudomonas* (7.5% of all NY sequences). The most often detected Betaproteobacteria genera in Florida were *Aquitalea* and *Burkholderia* (11.5% and 6% of FL sequences, respectively), but in New York, the most common Betaproteobacteria genera were *Undibacterium, Duganella*, and *Janthinobacterium* (5%, 5% and 4% of all NY sequences, respectively). The dominant Bacteroidetes genera in Florida clone libraries were *Nubsella*, *Lacibacter*, and *Filimonas*, accounting for 11, 7.5 and 7% of total Florida clones, respectively. Yet, in New York, the most dominant Bacteroidetes genera were *Chryseobacterium*, *Mucilaginibacter*, and *Pedobacter*, representing 15, 8 and 8% of all NY clones, respectively.

### The Impacts of Bacterial Consumers and other Members of the Aquatic Food Web (i.e., Insects) on Bacterial Community Structure

The non-bacterial key members of the pitcher plant food web were highly variable in abundance within pitcher plant leaves sampled within a bog and when community structure was compared between bogs, suggesting that 14 days from leaf opening is insufficient time for the communities to reach equilibrium [18], [29]. When all 30 leaves (15 within each site) were compared between sites, communities in New York contained significantly more resource input and higher protozoan densities and richness than communities in Florida (Table 2, Table 3; Mann-Whitney U Test: Resource input: p = 0.017, Protozoan Density p = 0.002; Protozoan Richness p = 0.0004). The abundances of mosquito larvae, midges and culturable bacteria were not significantly different between sites (Table 2, Table 3; Mann-Whitney U Test, Mosquito Larvae: p = 0.724; Midge p = 0.693; Culturable Bacteria Density p = 0.281).

**Table 2:** Community composition of New York phytotelmata.

Leaf	Water (ml)	Water clarity	Dead ants+ invert.^1^	*W. smithii* (instar) 1	*M. knabi* (midge) 1	Protozoan density^2^	Protozoan richness^2^	Culturable bacteria^3^	Culturable bacteria richness^3^
**1**	4	clear	0	1 (1^st^)	0	1,000	3	2,000	2
**2**	10	clear	2	0	0	7	2	322	2
**3**	5	clear	19	0	0	201	2	211	3
**4**	4	yellow	8	0	1	2,900	2	26,100	4
**5**	14	clear	5	16 (1^st^)	1	2	1	6,500	4
**6**	10	cloudy	35	7 (1^st^)	0	277	2	15,500	3
***Average*** ***Leaf 1–6***	*7.83* *SE +/−1.68*		*11.5 ants* *SE +/−5.43*	*4* *SE +/−2.64*	*0.333* *SE +/−0.210*	*730* *SE +/−457*	*2* *SE +/−0.258*	*8440* *SE +/−4240*	*2.83* *SE +/−0.401*
**7**	5	clear	6	0	0	111	1	1040	3
**8**	4	cloudy	1	1 (1^st^)	0	3890	2	305	3
**9**	10	clear	1	0	0	0	0	1900	4
**10**	2	clear	1 beetle	1 (1^st^)	0	0	0	634	2
**11**	4	cloudy	5+1 spider	0	0	1580	1	16,000	3
**12**	15	cloudy	9	2 (1^st^)	2	856	3	9,800	2
**13**	5	clear	2+1 fly	3 (1^st^)	3	7	2	15,400	4
**14**	3	cloudy	10	0	1	2	2	10,300	1
**15**	9	brownish	7+1 centipede	0	0	5	2	9,113	4
***Total Average***	*6.93* *SE +/−1.05*		*7.33 ants* *SE +/−2.36*	*2.07* *SE +/−1.11*	*0.533* *SE +/−0.236*	*722* *SE +/−309*	*1.67* *SE +/−0.232*	*7674* *SE +/−2033*	*2.87* *SE +/−0.256*

1 Other invertebg001rates observed within leaf water.

2 Density and Richness determined in a 0.1 ml aliquot of leaf water.

3 Density determined in a 0.1 ml aliquot of leaf water after a 10^−4^ dilution.

Community data (with the exception of bacterial sequences) collected for all 15 pitchers sampled in the New York bog. The first six samples were the samples also used for the RDP classification analysis.

**Table 3:** Community composition of Florida phytotelmata.

Leaf	Water (ml)	Water clarity	Dead ants+invert.^1^	*W. smithii* (instar) 1	*M. knabi* (midge) 1	Protozoan density^2^	Protozoan richness^2^	Culturable bacteria^3^	Culturable bacteria richness^3^
**1**	2.5	red	0+3 beetles	0	0	4	1	10,000	3
**2**	5	cloudy	0+1 spider	1 (2nd)	0	30	1	13,000	5
**3**	4.5	cloudy	22	1 (1^st^)	2	0	0	63,600	3
**4**	5	clear	1	9 (1^st^)	5	0	0	2,970	5
**5**	7	cloudy	3	0	3	20	1	27,800	3
**6**	1	clear	7	2 (1^st^)	0	0	0	4,000	4
***Average Leaf 1–6***	*4.17 SE+/−0.863*		*5.5 ants SE+/−3.47*	*2.16 SE+/−1.40*	*1.67 SE+/−0.843*	*9 SE+/−5.26*	*0.5 SE+/−0.224*	*20,200* *SE+/*−*9400*	*3.83* *SE+/−0.401*
**7**	3	clear	0	0	0	0	0	8,150	6
**8**	3	clear	0	2(4^th^), 1(1^st^)	0	0	0	6,500	4
**9**	5	clear	0	2(1^st^), 2(2^nd^)	0	0	0	4,050	4
**10**	4	clear	1 spider	0	0	0	0	6,000	3
**11**	4	clear	0	0	0	0	0	2,930	3
**12**	3	clear	0	0	0	1	1	10,500	5
**13**	2.5	dark red	5	0	0	0	0	32,200	3
**14**	4	clear	0	7 (1^st^)	0	0	0	8,900	5
**15**	4	clear	0	1(1^st^)	0	97	1	433	2
***Total Average***	*3.83 SE+/−0.380*		*2.53 ants SE+/−1.50*	*1.87 SE+/−0.723*	*0.667 SE+/−0.386*	*10.1 SE +/−6.61*	*0.333 SE+/−0.126*	*13,400 SE +/−4,260*	*3.87* *SE+/−0.291*

1 Other invertebrates observed within leaf water.

2 Density and Richness determined in a 0.1 ml aliquot of leaf water.

3 Density determined in a 0.1 ml aliquot of leaf water after a 10^−4^ dilution.

Community data (with the exception of bacterial sequences) collected in all 15 pitchers sampled in the Florida bog. The first six samples were the samples also used for the RDP classification analysis.

To relate the identification of specific bacterial groups in the pitchers of *S*. *purpurea* with the diversity and relative abundance of insect larvae and protozoans, we also analyzed the differences between pitchers for the six communities that were used to assess bacterial composition with SSU rRNA sequence libraries. We found that when these 6 communities were compared, New York pitcher plants contained qualitatively more, though not statistically significant, water than samples collected in Florida (Table 2, Table 3; One-way ANOVA, F = 3.76, p = 0.081) and had statistically significant higher protozoan densities and richness (Mann-Whitney U Test; p = 0.037 and p = 0.008, respectively). Of the 6 communities sampled within a site and analyzed based on RDP genus-level classification results, those communities with higher resource input, water level, protozoan density, or mosquito larvae presence did not have significantly different diversity, evenness or richness of bacterial genera.

## Discussion

Unlike the protozoans and metazoans that are part of the pitcher plant aquatic community, we found that at the beginning of the community succession, there was high variability in bacterial genera composition across small spatial scales (within a bog), as well as between bogs in NY and FL. Although sample size was small in this study, these observations are corroborated by previous work that used molecular techniques to identify bacteria in the *Sarracenia* system [11], [12], [13], [14]. Only a small percentage of bacterial genera were shared between *S. purpurea* leaves at both Florida and New York bog sites and bacterial community composition was more similar at the local scale. However, there was still great variation from pitcher to pitcher in phylogenetic structure as well as the relative abundance of genera.

At the phylum level, some lineages present in Florida were not found in New York (Firmicutes and Armatimondates); however, the phyla Proteobacteria and Bacteroidetes were the most abundant both within and between sites. These results are in congruence with Krieger and Kourtev [12], who sampled bacteria in *S. purpurea* leaves within Michigan bogs. This result highlights that large site differences in the bacteria in this system are found at the genus level, but certain phyla of bacteria may be important in the *S. purpurea* system across the plant’s geographic range. Bacteria from the phylum Bacteroidetes may be particularly important for this system as many of these organisms are well known for organic degradation and some are known to produce extracellular enzymes, likely necessary for processing nutrients for *S. purpurea*. The highly variable relative abundance of Bacteroidetes from leaf-to-leaf suggests however, that the selection pressure is not particularly strong and other environmental factors may impact pitcher leaf microbial communities.

Our study also attempted to understand how bacterial community composition is affected by other members of the aquatic food web. If the observed relationship between bacterial communities and the eukaryote structure of the remaining food web were to be described with classical food web theories (trophic cascade, predatory (top-down) and resource (bottom-up) control [30]; keystone predator [31]), we would expect that resources and food web structure would affect bacterial composition. For example, if insects falling into the pitchers were an important vector of transport for the bacteria and also an important resource (bottom-up control) for bacterial growth, the variation in the abundance and type of insect prey collected in a pitcher would be expected to impact the diversity and abundance of bacteria in each newly developing community. This effect could result either in a bacterial bloom or the continual colonization of new bacteria as a new prey enters the community.

If protozoans, rotifers, dipterans laying their eggs, and the resulting insect larvae, influenced bacterial composition through either transporting bacteria when entering the leaf or through internal community dynamics once inside the leaf, we would expect the presence of protozoans and rotifers, which prey on bacteria, to decrease bacterial abundance and affect bacterial diversity. The presence of top predator mosquito larvae would then release the bottom trophic level (bacteria) from predation pressure (top-down control, trophic cascade) and, by doing so, would increase the abundance [3] and influence bacterial diversity [11].

However, our data did not show these classical ecological properties of food web interactions (trophic cascade, predatory and resource control [30]; keystone predator [31]) when composition was compared between and among the two sites. It is important to note that a larger sample size that could be obtained with high-throughput sequencing would be needed in the future to fully determine the impact of the food web structure on bacterial composition in this system. From the data we present here, we found that, although bacterial composition was significantly different in pitchers in Florida compared to pitchers in New York, the abundance and variation of the top predator was the same at both sites. It therefore appears that the mosquito larvae in this system, which have been found to act as keystone predators [8] and to cause a trophic cascade [3], may not be affecting the difference in bacterial composition found between these two sites (but see [11]). Interestingly, we did find that the density and relative richness of protozoan species, which form the intermediate trophic level, was significantly different between the two sites. Therefore, the predation pressure of the intermediate trophic level, and possibly other non-tested factors, such as differences in habitat type, may cause the differences in genera composition between the two sites.

We conclude that although the non-bacterial members of this system have been found to exhibit similar properties and the same species throughout its geographic range [10], [17], the bacteria do not exhibit this same pattern, at least at the beginning of the season. This suggests one of two possibilities for community development. A strong environmental/ecological filter may exist for the non-bacterial trophic levels of this community, but not for the bacteria. Alternatively, the filter occurs later in community development for bacteria and interactions with the higher trophic level as well as resource input and extrinsic factors may shape the bacterial community in some aspects through time. Within the NY study site, this pattern is supported by findings in Gray [32], in which communities converge to be more similar in bacterial composition at the end of the season and to contain a different composition when compared to communities at the beginning of the season.

These data suggest that early in leaf community development specific bacterial species are not required by the plant to decompose trapped insects, and co-evolution between the plant and bacteria may not have occurred as it has for other members of this community (i.e., mosquito and midge larvae [33]). Furthermore, the variability observed in the pitchers, even within each sampling site, suggests that there are a number of factors influencing the leaf microbial community, including temperature, rainfall, pH, or deposition of bacteria through airborne particles or invertebrate vectors (e.g., carried in on the bodies of ants). As is true of soil microbial communities, microbial communities are diverse, with high functional redundancy, and the same is likely to be true of pitcher plants. Thus, while the plant and aspects of the food web may exert some selective pressure on the microbial community, these can be overcome by other factors out of their control.
